# Time series analysis of hemorrhagic fever with renal syndrome in mainland China by using an XGBoost forecasting model

**DOI:** 10.1186/s12879-021-06503-y

**Published:** 2021-08-19

**Authors:** Cai-Xia Lv, Shu-Yi An, Bao-Jun Qiao, Wei Wu

**Affiliations:** 1 Department of Epidemiology, School of Public Health, China Medical University, Shenyang, Liaoning China; 2 Liaoning Provincial Center for Disease Control and Prevention, Shenyang, Liaoning China

**Keywords:** Time series analysis, Hemorrhagic fever with renal syndrome (HFRS), XGBoost model, Multistep prediction

## Abstract

**Background:**

Hemorrhagic fever with renal syndrome (HFRS) is still attracting public attention because of its outbreak in various cities in China. Predicting future outbreaks or epidemics disease based on past incidence data can help health departments take targeted measures to prevent diseases in advance. In this study, we propose a multistep prediction strategy based on extreme gradient boosting (XGBoost) for HFRS as an extension of the one-step prediction model. Moreover, the fitting and prediction accuracy of the XGBoost model will be compared with the autoregressive integrated moving average (ARIMA) model by different evaluation indicators.

**Methods:**

We collected HFRS incidence data from 2004 to 2018 of mainland China. The data from 2004 to 2017 were divided into training sets to establish the seasonal ARIMA model and XGBoost model, while the 2018 data were used to test the prediction performance. In the multistep XGBoost forecasting model, one-hot encoding was used to handle seasonal features. Furthermore, a series of evaluation indices were performed to evaluate the accuracy of the multistep forecast XGBoost model.

**Results:**

There were 200,237 HFRS cases in China from 2004 to 2018. A long-term downward trend and bimodal seasonality were identified in the original time series. According to the minimum corrected akaike information criterion (CAIC) value, the optimal ARIMA (3, 1, 0) × (1, 1, 0)_12_ model is selected. The index ME, RMSE, MAE, MPE, MAPE, and MASE indices of the XGBoost model were higher than those of the ARIMA model in the fitting part, whereas the RMSE of the XGBoost model was lower. The prediction performance evaluation indicators (MAE, MPE, MAPE, RMSE and MASE) of the one-step prediction and multistep prediction XGBoost model were all notably lower than those of the ARIMA model.

**Conclusions:**

The multistep XGBoost prediction model showed a much better prediction accuracy and model stability than the multistep ARIMA prediction model. The XGBoost model performed better in predicting complicated and nonlinear data like HFRS. Additionally, Multistep prediction models are more practical than one-step prediction models in forecasting infectious diseases.

**Supplementary Information:**

The online version contains supplementary material available at 10.1186/s12879-021-06503-y.

## Background

Hemorrhagic fever with renal syndrome (HFRS) is a zoonotic disease caused by hantaviruses that cause a high degree of harm to humans. To date, more than 28 hantaviruses resulting in human diseases have been identified worldwide. Most HFRS cases occur in Asian and European countries, such as China, South Korea and Russia. More than 100,000 cases of HFRS occur every year worldwide, and China accounts for more than 90 % of them [1, 2]. In recent years, the number of HFRS cases in mainland China has shown an overall downward trend [3], but it is still prevalent in some regions, such as Heilongjiang, Liaoning, Jilin, Shandong, Shanxi and Hebei provinces [4]. It should be pointed out that epidemic areas for rodent have a tendency to spread towards cities, as hantavirus is carried and spread by rodents. The main transmission routes from rodents to humans are aerosolized excreta inhalation and contact infection. Person-to-person spread may occur but is extremely rare [3–5]. The clinical symptoms of HFRS are mainly characterized by fever, hemorrhaging and kidney damage with a 4 to 46 day incubation period [5]. HFRS can lead to death if the patient is not treated in time. The Chinese Center for Disease Control (CDC) established a surveillance system for HFRS in 2004 and classified it as a class II infectious disease. The surveillance system requires newly confirmed cases of HFRS to be reported within 12 h, which ensures the accuracy and timeliness of the data [6]. Although the government and health departments have taken on many control measures, such as active rodents control, vaccination implementation, health education implementation, environmental management of the epidemic areas, and disease surveillance strengthening, HFRS still severely affects people’s health with approximately 9,000–30,000 cases annually in China [7].

To delineate the changing trend in the incidence of infectious diseases, domestic and foreign researchers have applied various statistical and mathematical models to the prediction of infectious diseases, such as random forest [8], gradient boosting machine (GBM) [9] and support vector machine models [10]. At present, some models have been used in predicting HFRS, including neural networks [11] and generalized additive models (GAMs) [12]. Most of these methods are based on one-step forecasting. The autoregressive integrated moving average (ARIMA) model, as a fundamental method in time series analysis that regresses the lag value of the time series and random items to build a model, has been applied in many fields [13]. Although an ARIMA model can capture the linear characteristics of infectious disease series well, such as the autoregressive (AR) term and moving average(MA) term, some information may be lost when it analyzes the residuals consisting of non-linear information [14]. XGBoost is a boosting algorithm based on the evolution of gradient boosting decision tree (GBDT) algorithm, which has achieved remarkable results in practical applications due to its high accuracy, fast speed and unique information processing scheme. Compared with traditional statistical models, it has advantages in predicting nonlinear data [15–19]. Previous studies usually applied one-step predictive statistical models to characterize and predict epidemic trends in infectious diseases. Currently, a multistep XGBoost model has not been used to forecast infectious diseases such as HFRS.

In this study, we aim to develop a prediction model for HFRS in mainland China by using one-step and multistep XGBoost models and comparing them with an ARIMA model.

## Methods

### Data collection

We collected HFRS incidence data from 2004 to 2018 from the official website of the National Health Commission of the People’s Republic of China (http://www.nhc.gov.cn). Based on the requirements of China’s Infectious Disease Control Law, hospital physicians must report every HFRS case within 12 h to the local health authority. Once the patient is diagnosed with a suspected case based on clinical symptoms, patient blood samples are collected and sent to local CDC laboratories for serological confirmation; if the result is positive, it is considered as a confirmed case. Local health authorities later report monthly HFRS cases to the national health department for surveillance purposes. However, the monitoring system relies on hospitals passively monitoring the occurrence of infectious diseases, and there will be a certain time delay in information collection. If the patient’s symptoms are mild and not require hospitalization, underreporting may occur [20]. The dataset analyzed during the study is included in Supplementary Material 1. The HFRS data from 2004 to 2017 were adopted to establish the seasonal ARIMA model and XGBoost model, while the 2018 data were used for model verification.

### ARIMA model

An ARIMA model is a time series forecasting method that was first proposed by Box and Jenkins in 1976 [21]. The principle of the ARIMA model is to adopt appropriate data conversion to transform nonstationary time series into stationary time series and then adjust the parameters to find the optimal model. Finally, the changes in past trends are quantitatively described and simulated to predict future outcomes [13, 22]. The specific procedures for establishing the seasonal ARIMA model were as follows: first, we performed a Box-Cox transformation to smooth the variance of the original HFRS time series. Simultaneously, long-term trends and seasonal differences were stabilized through first-order differences and seasonal differences. Then, we preliminarily judge the possible parameter values of the ARIMA model based on the truncation and tailing properties of the autocorrelation function (ACF) and partial autocorrelation function (PACF) diagrams. The advantages and disadvantages of the model fit were evaluated by the corrected Akaike information criterion (CAIC) value, and the model with the smallest CAIC value was considered the optimal model. After the order of the specific parameters was determined, a parameter test was performed through maximum likelihood estimation (MLE). Finally, the Ljung-Box test judges whether the residual sequence is white noise.

### Building the XGBoost model

XGBoost, a kind of boosting algorithm, which assembles multiple learning algorithms to achieve a better predictive performance than any of the constituent learning algorithms alone, has excelled in many fields. Compared with the traditional GBDT algorithm, XGBoost applies a second-order Taylor expansion to the loss function and simultaneously implements the first derivative and the second derivative. In addition, a regularization term is added to the objective function, which improves the generalizability of a single tree and reduces the complexity of the objective function. In short, XGBoost has attracted the attention of researchers due to its fast speed, excellent classification effect, and ability to allow custom loss functions.

The classification and regression tree (CART) algorithm, first proposed by Breiman et al., refers to the general term of a classification tree and regression tree. The CART classification tree introduces the Gini coefficient to replace the information gain or information gain rate. The regression tree adopts different methods to evaluate the effect, including the prediction error (mean squared error, log error, etc.). Therefore, the node is no longer a category but a numerical value. In a CART model, for any feature j, there is a corresponding segment point s. If j is less than s, it is divided into the left-hand subtree. Otherwise, it is divided into the right-hand tree, as in formula (1).

1 $${R}_{1}\left(j,s\right)=\left\{x|{x}^{\left(j\right)}\le s\right\} \,and \,{R}_{2}\left(j,s\right)=\left\{x|{x}^{\left(j\right)}>s\right\}$$

The objective function of a typical CART regression tree is defined in formula (2):

2 $$\sum _{xi\in Rm}({yi-f\left(xi\right))}^{2}$$

As shown in formula (3), find the corresponding j and s that minimize the MSE of c1 and c2, respectively, and minimize the sum of the MSE between the two parts of c1 and c2. When we traverse all the segment points s of all features j, we can find the optimal j and s, and finally obtain a regression tree.

3 $$\underset{\text{j},\text{s}}{\text{min}}\left[\underset{{\text{x}}_{\text{i}}\in {\text{R}}_{1}(\text{j},\text{s})}{\text{min}}{(\text{y}\text{i}-\text{c}1)}^{2}+\underset{{\text{x}}_{\text{i}}\in {\text{R}}_{1} (\text{j},\text{s})}{\text{min}}{(\text{y}\text{i}-\text{c}2)}^{2}\right]$$

$${\widehat{ \text{c}}}_{1}=\text{a}\text{v}\text{e}\left({\text{y}}_{\text{i}}\mid {\text{x}}_{\text{i}}\in {\text{R}}_{1}(\text{j},\text{s})\right)$$

4 $${\widehat{\text{c}}}_{2}=\text{a}\text{v}\text{e}\left({\text{y}}_{\text{i}}\mid {\text{x}}_{\text{i}}\in {\text{R}}_{2}(\text{j},\text{s})\right)$$

The CART regression tree applies the mean or median of the final leaves to predict the output. To avoid overfitting, cost complexity pruning (CCP) is used to prune the non-leaf node with the smallest error gain and delete the child nodes with the non-leaf node.

The XGBoost algorithm is mainly composed of two parts: the decision tree algorithm and gradient boosting algorithm. Gradient boosting is an excellent technique for constructing prediction models and a representative algorithm for boosting. The theory of boosting is to establish weak evaluators individually and iteratively integrate multiple weak evaluators. The gradient boosting tree uses the CART algorithm as the main structure. Therefore, the steps of the XGBoost algorithm can be expressed as follows (formular (5)):

5 $$\widehat{{\text{y}}} = \phi \left( {{\text{x}}_{{\text{i}}} } \right) = \sum\limits_{{{\text{k}} = 1}}^{{\text{K}}} {{\text{f}}_{{\text{k}}} } \left( {{\text{x}}_{{\text{i}}} } \right)$$

In the XGBoost model, every leaf node has a forecasting score, called the leaf weight. ${f}_{k}\left({x}_{i}\right)$ is the value of all samples on this leaf node, where represents the th decision tree and represents the feature vector of sample. Each tree was added iteratively to keep the predicted value $\hat {{y}}_{i}$ as close as possible to the actual value y_i_. Therefore, the following function reaches the minimum after t iterations:

6 $$O\text{b}{\text{j}}^{\left(\text{t}\right)}=\sum _{\text{i}=1}^{\text{n}} \text{l}\left({\text{y}}_{\text{i}},{\widehat{\text{y}}}_{\text{i}}^{(\text{t}-1)}+{\text{f}}_{\text{t}}\left({\text{x}}_{\text{i}}\right)\right)+{\Omega }\left({\text{f}}_{\text{t}}\right)+\text{ constant }$$

As shown in formula (6), the objective function consists of two parts: a loss function and a regularization term. The loss function assesses the forecasting function of the XGBoost model on the training data, and the regularization term ${\Omega }\left({f}_{t}\right)$ prevents the model from being too complicated. $\hat {{\mathbf{y}}}^{(t-1)}$ is the predicted value of the last iteration and ${\text{f} }_{t}$is a new function that the model learns. Next, a second-order Taylor development of the error term was performed on the objective function. Then the first derivative and the second derivative are defined as follows:

7 $${\text{Obj}}^{({\rm t})}\simeq \sum _{{\text{i}}=1}^{{\text{n}}} \left[{\text{l}} \left({\text{y}}_{\text{i}}, {\widehat{\text{y}}}_{\text{i}}^{({\text{t}}-1)}\right)+{{\text{g}}_{\text{i}}}{{\text{f}}_{\text{t}}}\left({\text{x}}_{\text{i}}\right)+\frac{1}{2}{{\text{h}}_{\text{i}}}{{\text{f}}_{\text{t}}^{2}}\left({\text{x}}_{\text{i}}\right)\right]+{\Omega }\left({\text{f}}_{\text{t}}\right)+{\text{constant}}$$

8 $${\text{g}}_{\text{i}}={\partial }_{{\widehat{\text{y}}}^{(\text{t}-1)}}\text{l}\left({\text{y}}_{\text{i}},{\widehat{\text{y}}}^{(\text{t}-1)}\right), {\text{h}}_{\text{i}}={\partial }_{{\widehat{\text{y}}}^{(\text{t}-1)}}^{2}\text{l}\left({\text{y}}_{\text{i}},{\widehat{\text{y}}}^{(\text{t}-1)}\right)$$

First, we define the mapping function of the decision tree: q indicates the structure of the tree, and w is the leaf node weight vector (the value of the sample predicted by the model).

9 $${f}_{t}\left(x\right)={w}_{q\left(x\right)},w\in {\text{R}}^{T},q:{\text{R}}^{d}\to \{\text{1,2},\cdots ,T\}$$

The complexity of the XGBoost tree is shown in formula (10). T is the quantitative complexity of leaf nodes in the tree, and the sum of squares term represents the L2 regularization term of the leaf node.

10 $${\Omega }\left({f}_{t}\right)=\gamma T+\frac{1}{2}\lambda \sum _{j=1}^{T} {w}_{j}^{2}$$

After combining the defined loss function and complexity of the tree, the objective function can be expressed by formula (13).

11 $$Ob{j}^{\left(t\right)}=\sum _{j=1}^{T} \left[\left(\sum _{i\in {I}_{j}} {g}_{i}\right){w}_{j}+\frac{1}{2}\left(\sum _{i\in {I}_{j}} {h}_{i}+\lambda \right){w}_{j}^{2}\right]+\gamma T$$

12 $${G}_{j}=\sum _{i\in {I}_{j}} {g}_{i}, {H}_{j}=\sum _{i\in {I}_{j}} {h}_{i}$$

13 $$Obj=-\frac{1}{2}\sum _{j=1}^{T} \frac{{G}_{j}^{2}}{{H}_{j}+\lambda }+\gamma T$$

Because it is not possible to traverse all the tree structures, constructing a decision tree based on space division is an NP problem. XGBoost uses a greedy algorithm to traverse the segmentation points of all features in the CART regression tree and calculates the gain before and after the segmentation point to determine whether a node continues to grow. The node will split when the value of the objective function after splitting is higher than the gain of the single-leaf node. At the same time, the maximum depth of the tree and a threshold should be set to limit its growth. The gain formula is shown in formula (14):

14 $$\text{Gain }=\frac{1}{2}\left[\frac{{G}_{L}^{2}}{{H}_{L}+\lambda }+\frac{{G}_{R}^{2}}{{H}_{R}+\lambda }-\frac{{\left({G}_{L}+{G}_{R}\right)}^{2}}{{H}_{L}+{H}_{R}+\lambda }\right]-\gamma$$

One-hot encoding was used to address the seasonality. Three types parameters should be set when building the XGBoost model: general parameters, booster parameters and task parameters. The XGBoost model also draws on the idea of random forest, introducing row sampling and column sampling that can reduce the amount of calculation and prevent overfitting. Moreover, it introduces the early-stopping mechanism to prevent overfitting. In this study, the booster parameter is gbtree; early_stopping_round was set to 5; subsample and colsample_bytree were set from 0.3 to 0.7; max_depth was set to 2 and 3; min_child set to 1 and 2, the learning rates of XGBoost were set to 0.04, 0.05 and 0.06; and eval_metric was set to ‘rmse’. A grid search was conducted to exhaustively search for specified parameter values when the potential parameter values were ordered and combined. Notably, the performance of the XGBoost was evaluated by tenfold cross-validation and the RMSE. Additionally, XGBoost can rank the importance of variables by the frequency functions used to split the feature. After the XGBoost model was built, the accuracies of the one-step forecast and multistep forecast were compared by the RMSE, MAE and MAPE.

### One-step forecasting and multistep forecasting

Generally, a one-step time series uses actual historical data, such as data at time t-n, time t-(n-1), time t to predict the value at time t + 1 in the next step. In contrast, when performing multistep prediction, single-step prediction is performed and the predicted value is used (instead of the actual value) as an input variable for the second step of prediction. Then, the process was repeated until all the predicted values were obtained [23, 24]. There are four multistep forecasting strategies: direct forecasting, recursive forecasting, direct recursive hybrid forecasting and multioutput forecasting. One-step forecasting is more accurate, but it will prevent the model from simulating the trends in the next month. When the forecast cycle is long, a multistep forecast is prone to face larger error accumulation. When the forecasted value is used as input, the error will inevitably accumulate with the input value in the next step. In this study, one-step forecasting and multistep forecasting were carried out.

### Model comparison and data analysis

Model evaluation and comparison are mainly judged by the accuracy of the model. The accuracy refers to the degree to which the predicted result matches with the actual result, so the error can be used to evaluate the accuracy of the prediction model. The smaller the error is, the better the fitting effect. Model evaluation generally includes two parts: training sample evaluation and prediction sample evaluation. To better compare the accuracy of the ARIMA and XGBoost models, a series of evaluation indices were applied in this study. mean error (ME), root mean squared error (RMSE), mean absolute error (MAE), mean percentage error (MPE), mean absolute percentage error (MAPE), mean absolute scaled error (MASE) and autocorrelation of errors at lag 1 (ACF1). Generally, the larger the criteria are, the greater the error size is. Theil’s U statistic measures the accuracy by comparing the predicted results with the prediction results using minimal historical data. It tends to place more weight on large errors by squaring the deviations and overstating errors, which can help eliminate methods with large errors. Theil’s U < 1 indicates that the predicted results are better than the expected results.

The HFRS data analysis process was completed in R version 3.6.2. Packages like TSstudio, forecast, xgboost were included to achieve different functions. In addition, we set the statistical significance level at 0.05.

## Results

### ARIMA model

**Fig. 1:**
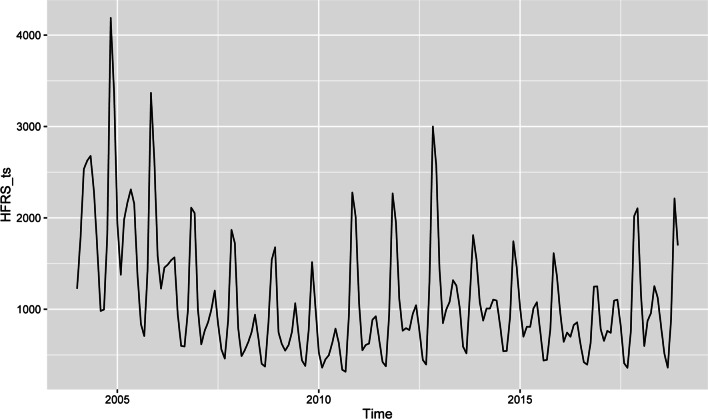
Time series plot for cases of HFRS in mainland China from January 2004 to December 2018

**Fig. 2:**
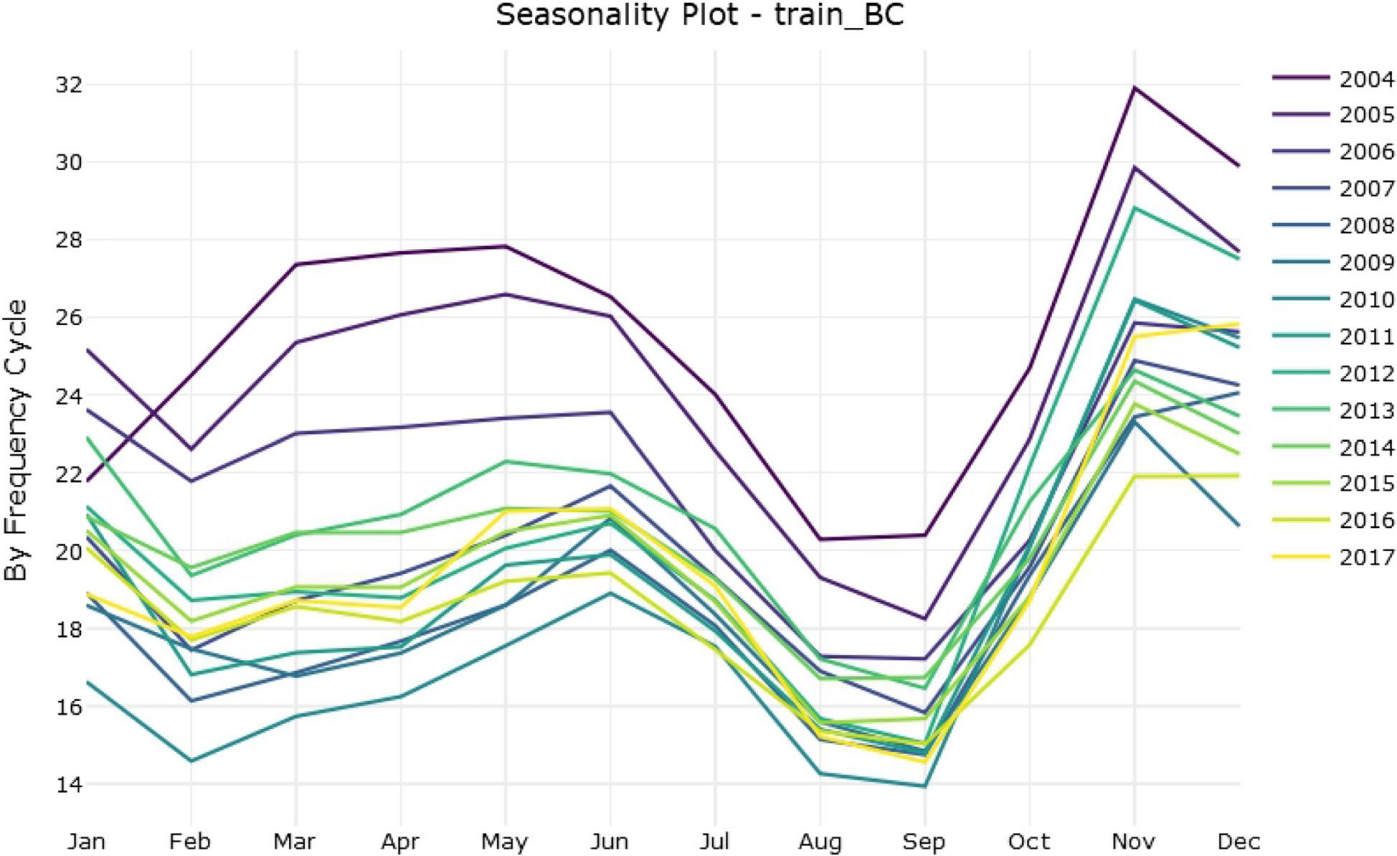
Monthly chart of HFRS cases from 2004 to 2017

**Fig. 3:**
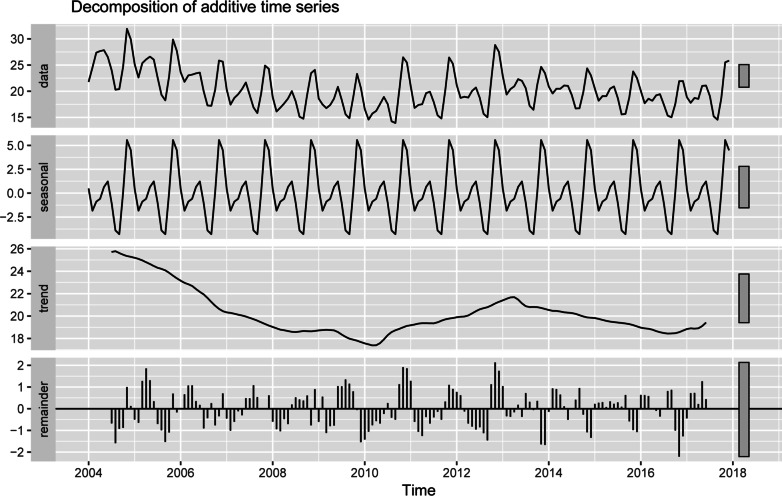
Seasonal decomposition of the Box-Cox-transformed HFRS cases

As shown in Fig. 1, the original time series graph showed a slight downward trend and seasonal variation. The number of HFRS cases had a bimodal seasonal distribution throughout the year (Fig. 2), one from October to January of the following year and the other from March to June, which means that the time series was not stationary. Therefore, logarithmic or square root conversion was used to transform the time series variance. The time series diagram after applying a Box-Cox transformation is shown in Fig. 3. The small gray blocks of different sizes show the proportion of each component. The additive time series decompositions subjected to Box-Cox transformation were arranged in order of magnitude, including the original data, season, trend and noise element. The seasonal component showed obvious periodicity, while the trend showed an overall decrease from 2004 to 2010 but increased briefly in 2010–2013. In addition, there was no noticeable form of noise.

**Fig. 4:**
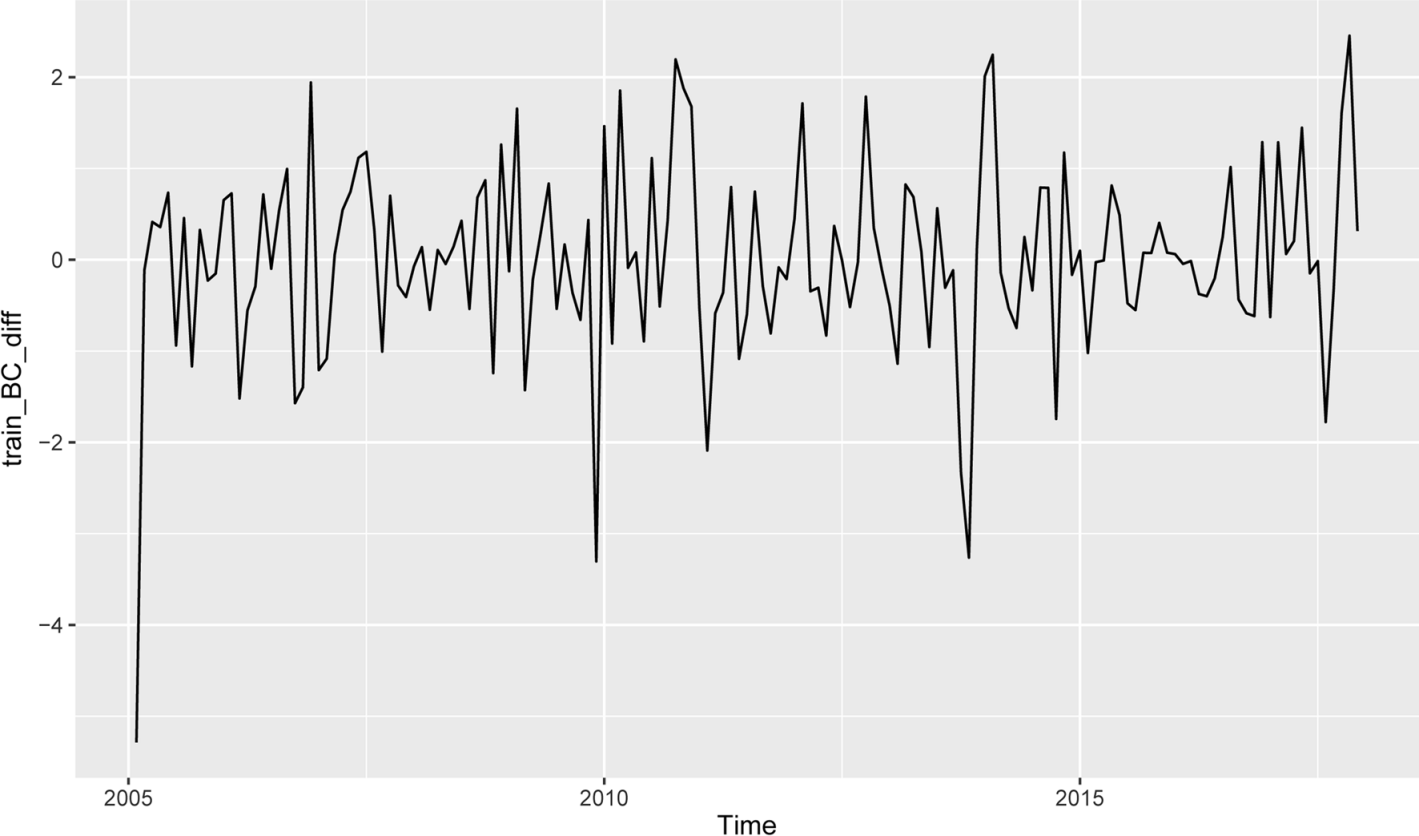
Plot of the Box-Cox-transformed HFRS cases

To eliminate seasonal characteristics and long-term trends in the time series, the first difference (d = 1) and seasonal difference (D = 1) were used (Fig. 4). The ADF test demonstrated that the time series after the difference was stable (t =− 6.4674, p < 0.01). Consequently, from d = 1 and s = 12, the seasonal ARIMA model can be preliminarily denoted by ARIMA (p, 1, q) × (P, 1, Q)_12_.

**Fig. 5:**
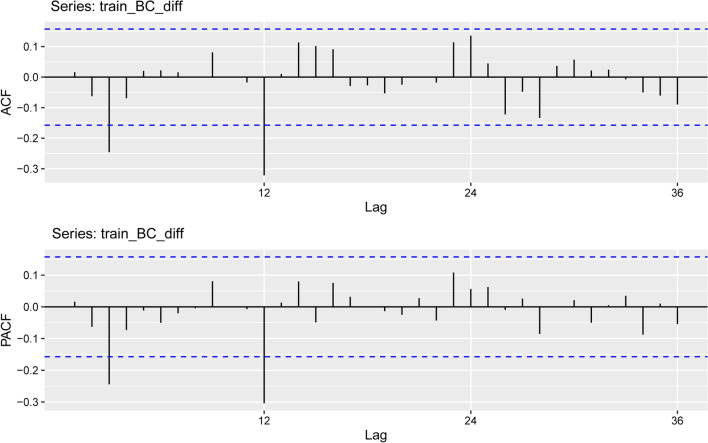
Autocorrelation and partial autocorrelation plots of the differenced HFRS incidence series

**Fig. 6:**
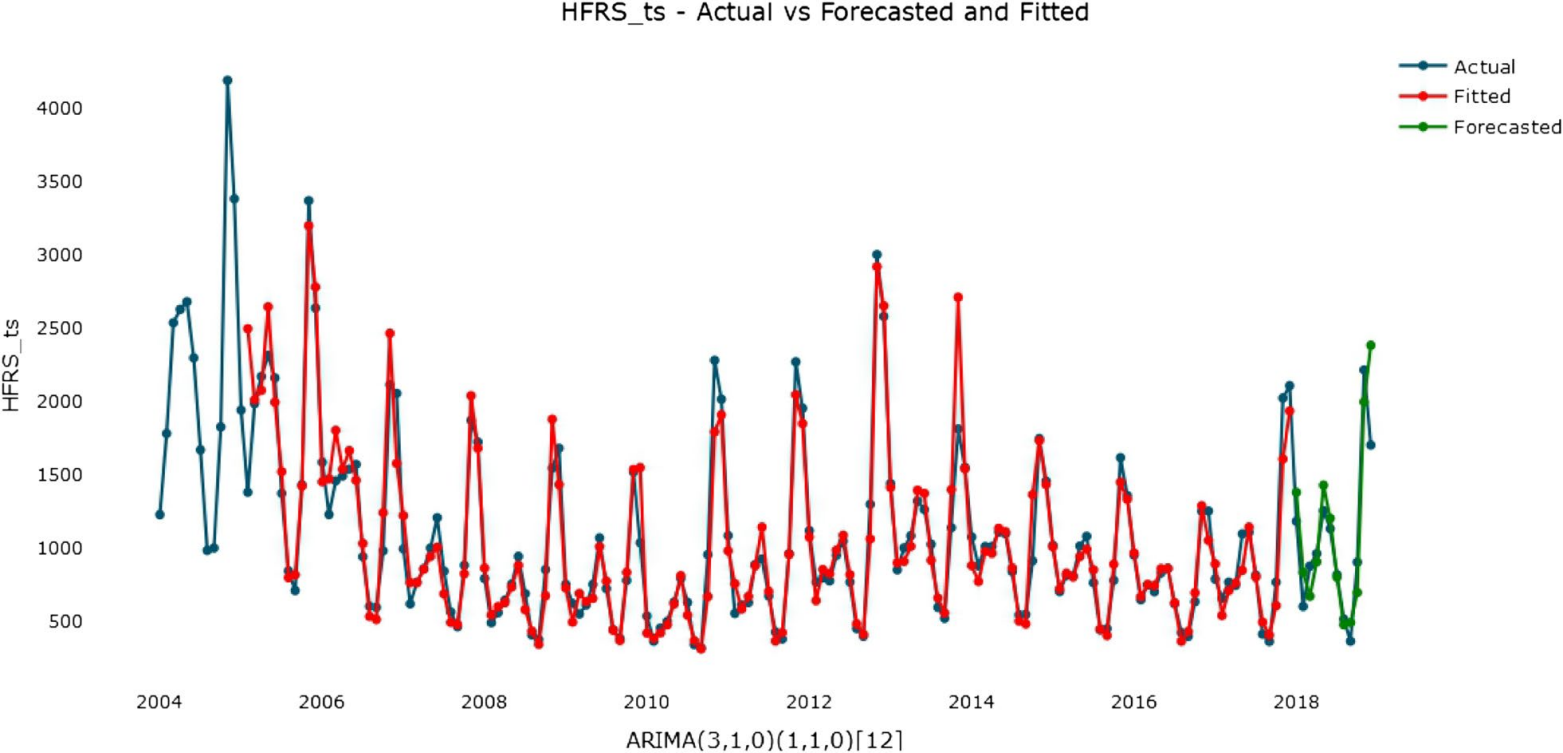
The curves of the fitted ARIMA model, forecasted ARIMA model and actual HFRS incidence series

As seen in the graphs of the ACF and PACF (Fig. 5). The ACF had obvious peaks at lags 3 and 12, indicating respectively nonseasonal MA (3) components and seasonal MA (1) components respectively. In addition, in the PACF graph, the obvious lag peaks at 3 and 12 indicate a nonseasonal AR (3) element and a seasonal AR (1) element. Therefore, the parameters were set as follows: p from 0 to 3, q from 0 to 3, P from 0 to 1 and Q from 0 to 1. By assembling all possible values of each parameter, multiple candidate models are generated. Nine models remained after the residual and parameter test was implemented, and the ARIMA (3, 1, 0) × (1, 1, 0)_12_ model had the smallest CAIC (427.1528) (Table 1). The Ljung–Box test (Q = 7.5588, p = 0.9944) indicated that the sequence residual was white noise, which means that the final fitted data sequence was stationary. The estimated parameters of the ARIMA (3, 1, 0) × (1, 1, 0)_12_ model are listed in Table 2. The curves of training, forecasting and the actual HFRS incidence by ARIMA model are pictured in Fig. 6.

**Table 1:** CAIC value and Ljung-Box Q value of the candidate seasonal ARIMA models

Model	CAIC	Ljung-Box Q	P value
ARIMA (0,1,3) × (1,1,1)_12_	429.244	7.091	0.994
ARIMA (0,1,3) × (1,1,0)_12_	427.345	7.429	0.995
ARIMA (0,1,3) × (0,1,1)_12_	428.220	12.07	0.914
ARIMA (3,1,0) × (1,1,1)_12_	429.108	7.347	0.992
ARIMA (3,1,0) × (1,1,0)_12_	427.154	7.559	0.994
ARIMA (3,1,0) × (0,1,1)_12_	427.666	12.552	0.896
ARIMA (3,1,3) × (1,1,1)_12_	430.864	7.068	0.972
ARIMA (3,1,3) × (1,1,0)_12_	428.906	7.333	0.979
ARIMA (3,1,3) × (0,1,1)_12_	429.528	12.340	0.779

**Table 2:** Estimated parameters of the seasonal ARIMA **(**3,1,0) × (1,1,0)_12_ model

Model parameter	Estimate	Standard error	95 % CI of the estimate
AR3	− 0.311	0.087	(− 0.481, − 0.142)
Seasonal AR1	− 0.405	0.082	(− 0.565, − 0.245)

### XGBoost model

**Fig. 7:**
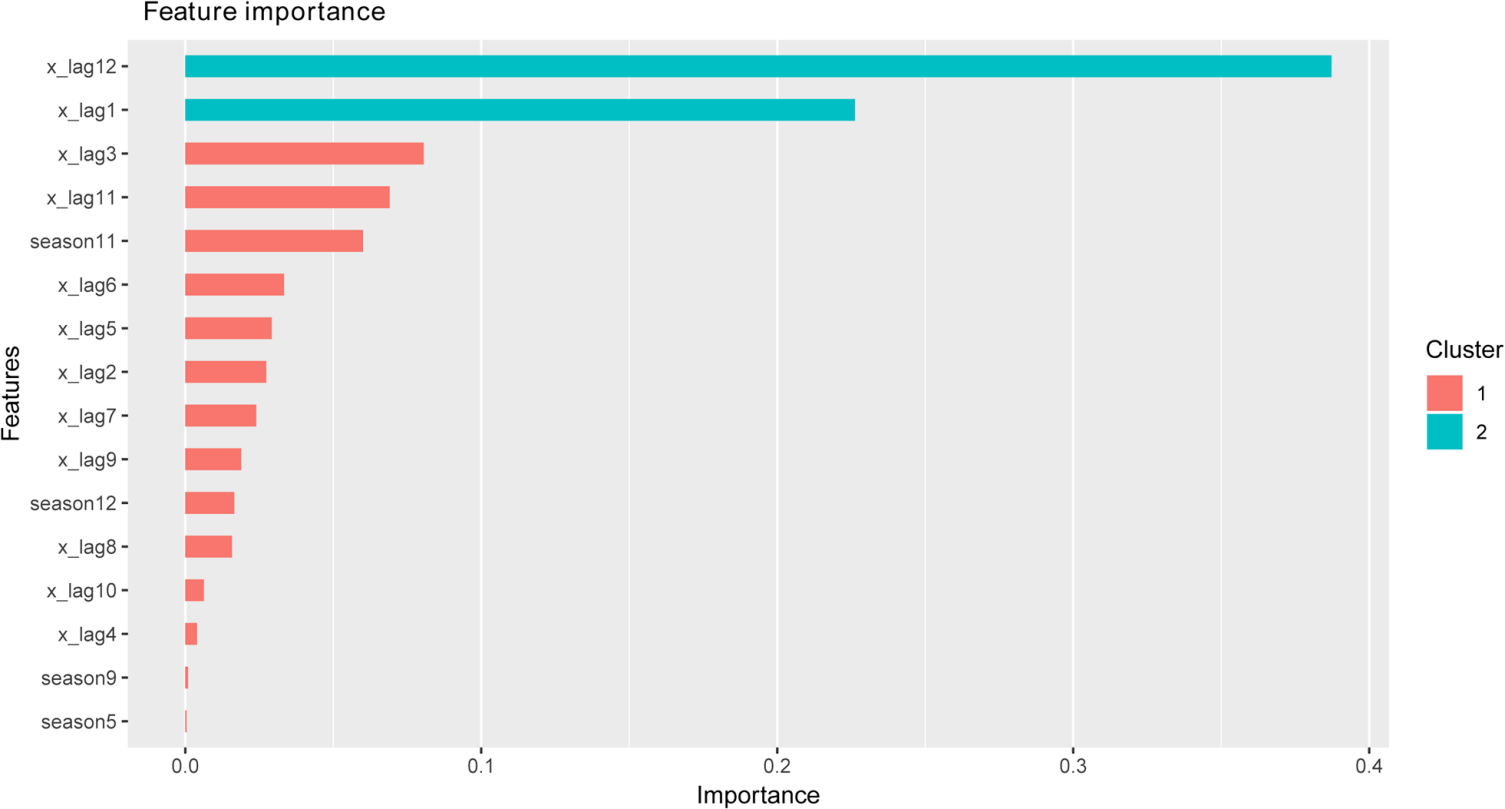
Importance of the XGBoost characteristic variables

**Fig. 8:**
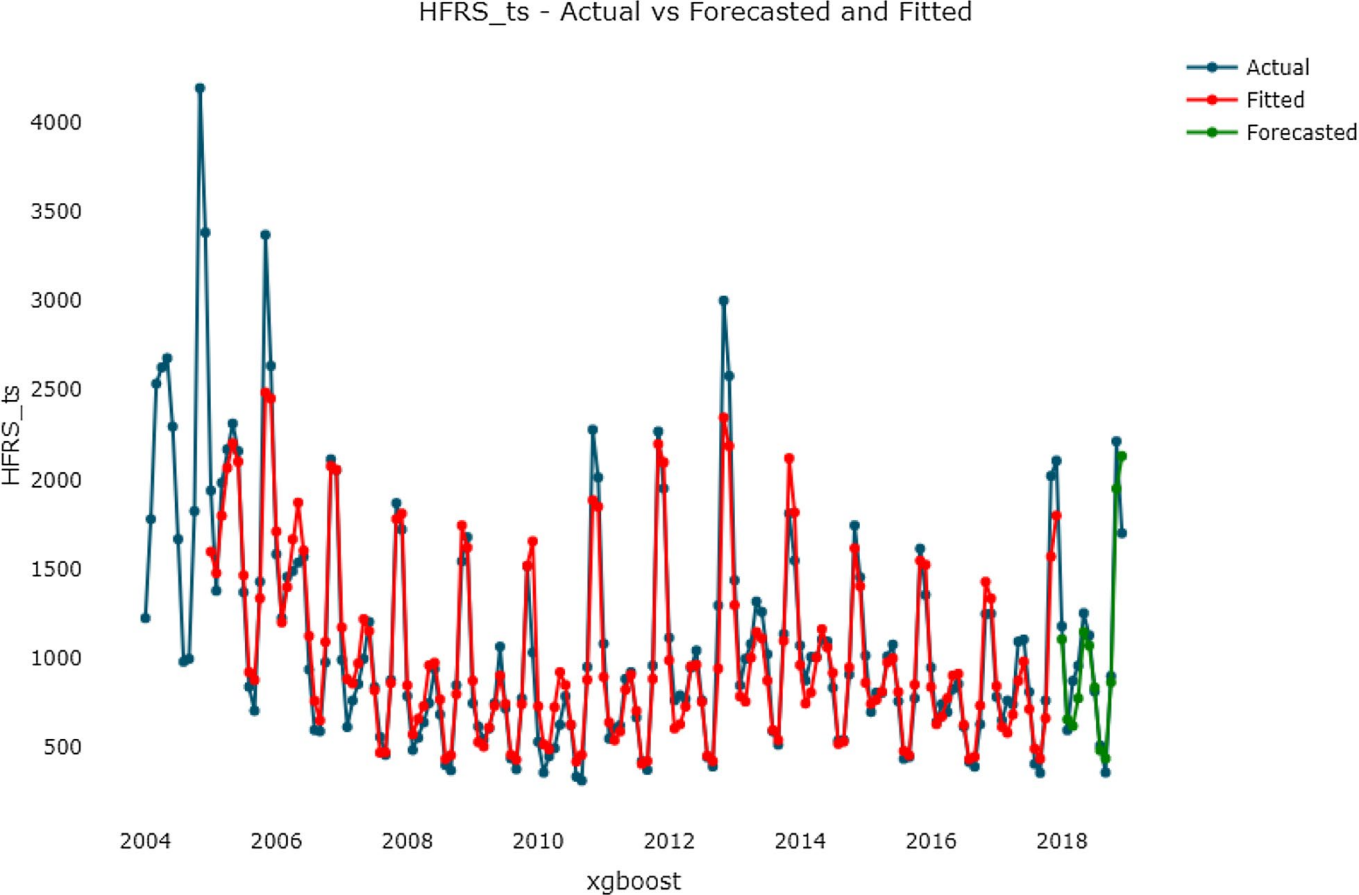
The curves of the fitted XGBoost model, forecasted XGBoost model and actual HFRS incidence series

The grid search algorithm was used in the XGBoost model to realize the automatic optimization of the parameters. In this research, we realized automatic optimization of max_depth, n_estimators and min_child_weight. According to the grid search and tenfold cross-validation, the possible parameters are shown in Table 3. Among all six combined parameters, the first had the lowest test RMSE (238.3084). The optimal parameters of the XGBoost model were listed in Table 4. The importance of a feature is determined by whether the forecasting capability changes significantly when the feature is replaced by random noise. In the XGBoost algorithm, we input several features to calculate the feature importance and determine how each feature contributes to the prediction performance in the training step (Fig. 7). Characteristic variables such as x_lag12 and x_lag1 had a significant impact on the prediction of the number of HFRS cases. Finally, based on the hyperparameter optimization results, the final one-step forecasting model was built. The curves of training, forecasting and the actual HFRS incidence by the XGBoost model are showed in Fig. 8.

**Table 3:** Possible parameters of the XGBoost model

Model Parameters	Best Rounds	Test RMSE	Train RMSE	SubSamp Rate	ColSamp Rate	Depth	Eta	MinChild
1	105	238.308	161.126	0.400	0.600	2	0.050	2
2	113	238.591	160.885	0.400	0.400	2	0.050	2
3	96	239.072	155.984	0.400	0.500	2	0.060	2
4	95	239.153	154.751	0.400	0.600	2	0.060	2
5	133	239.843	138.405	0.600	0.300	2	0.050	1
6	179	239.886	136.431	0.600	0.300	2	0.040	2

**Table 4:** List of the optimal parameters and description of the XGBoost model

Parameters	Value
Booster	‘gbtree’
Objective	‘reg: squared error’
Early_stopping_rounds	5
Eval_metric	‘rmse’
Min_child_weight	2
Subsample	0.4
Colsample_bytree	0.6
Eta	0.05
Nrounds	200
Depth	2

#### Comparison of the models

Table 5 shows the one-step and multistep forecasting accuracies of the two models. In the training sample, the ME, MAE, MAPE, MPE and MASE of XGBoost were higher than those of the ARIMA model, whereas the RMSE of XGBoost was lower than that of the ARIMA model. In the test sample, the ME, RMSE, MAE, MPE, MAPE and MASE of XGBoost model were obviously lower than those of the ARIMA model in both one-step forecasting and multistep forecasting. Therefore, the XGBoost model had a better forecasting performance in the prediction of the number of HFRS cases.

**Table 5:** The one-step and multistep forecasting accuracy of the ARIMA and XGBoost models

Model Strategy Index	ARIMA				XGBoost			
	One-step		Multistep		One-step		Multistep	
	Training set	Test set	Training set	Test set	Training set	Test set	Training set	Test set
ME	− 7.149	− 61.448	− 7.149	− 259.878	8.111	33.622	8.111	97.931
RMSE	181.977	249.276	181.977	302.781	166.311	178.547	166.311	223.187
MAE	108.160	185.367	108.160	259.878	113.219	132.055	113.219	173.403
MPE	− 0.937	− 6.575	− 0.937	− 30.121	− 2.403	2.383	− 2.403	6.348
MAPE	10.293	18.561	10.293	30.121	11.596	12.353	11.596	15.615
MASE	0.442	0.757	0.442	1.062	0.462	0.526	0.462	0.691
ACF1	0.016	− 0.169	0.016	− 0.159	0.424	− 0.232	0.424	− 0.047
Theil’s U	NA	0.375	NA	0.441	NA	0.273	NA	0.398

## Discussion

This study showed the seasonal distribution of HFRS cases. The main incidence peaks were concentrated from October to January, especially in November of the following year. The second incidence peak occurred from March to June. The overall shape was bimodal, which was consistent with the literatures reported in the South Korean army and different regions of China [4, 25]. In addition, the incidence of HFRS in mainland China from 2004 to 2018 showed an overall downward trend, but there was a clear upward trend in 2010 that continued until 2013. The periodicity of HFRS incidence may be related to climate factors, the number of rodents in the wild, and the accumulation speed of susceptible people. As an important climate factor, the monsoon phenomenon may affect periodic trends in HFRS, which can change annually. Since the data collected in this study were not from a sufficiently long period, the periodicity was not obvious in this study. The influence of meteorological factors and monsoon phenomena on HFRS can be considered in the future.

Therefore, understanding the changing trend in HFRS is particularly important for exploring the influencing factors. It is also crucial for predicting epidemics and formulating corresponding preventive and early-warning measures. The accuracy of infectious disease forecasting has drawn the attention of a number of scholars [9, 26, 27]. Many mathematical methods and statistical models have been applied to predict HFRS incidence. The ARIMA model is developed based on a linear regression model, combining the advantages of autoregressive and moving average models, which can explain the data well. We can obtain the coefficient of each variable and know whether each coefficient is statistically significant. Stationary data are a prerequisite for establishing an ARIMA model; thus, the seasonal ARIMA model needs to transform nonlinear data into linear data after differencing and transformation. According to the characteristics of HFRS, we decomposed the infectious disease time series into trend components, seasonal components and random fluctuation components. The more differences use, the more data are lost. In this study, the first-order and 12th-order differences were used, so 13 months of data were lost. When forecasting, the ARIMA model considers only historical data to understand the disease trend and obtain a more accurate prediction effect instead of requiring specific influencing factors. Therefore, the ARIMA method is easy to master and widely used. However, the nonlinear mapping performance of ARIMA models is weak, and its accuracy is unsatisfactory when it tries to fit and predict nonlinear and complex infectious disease time series. For example, in this study, the fitting effect was not perfect when the disease trend changed suddenly, and the error between the fitted value and the actual value in May 2010 and 2013 was relatively large (Fig. 6). Many factors can affect HFRS, including meteorological factors and human-made control measures, most of which have a nonlinear relationship with the number of cases, so when the number of HFRS suddenly increases or decreases, these nonlinear factors may affect the fitting accuracy of the ARIMA model. In addition, the ARIMA method is more suitable to predict a short-term time series. Thus, it is necessary to constantly collect data and obtain the longest time series possible. Based on the characteristic of the ARIMA model and HFRS, this study used the monthly incidence data of HFRS from 2004 to 2017 to establish a seasonal ARIMA model. The results showed that the ARIMA (3, 1, 0) × (1, 1, 0)_12_ model can better fit and predict the monthly incidence than other forms.

The XGBoost model is a powerful machine learning algorithm, especially in terms of the speed and accuracy are concerned. It is good at dealing with nonlinear data but has poor interpretability. From studies in other fields, the XGBoost model performed well in predicting nonlinear time series [28–31]. By integrating multiple CART models, XGBoost model can achieve a better generalizability than a single model, which means that the XGBoost has a larger postpruning penalty than a GBDT model and makes the learned model less prone to overfitting. Moreover, a regularization term is added to control the complexity reduce the variance of the model. Moreover, XGBoost model is a hyperparameter model [32], that can control more parameters than other models and is flexible to tune parameters. Compared with the complexity of the conditions that the ARIMA model needs to meet, the modeling process of the XGBoost is very simple. In this study, a grid search was conducted to exhaustively search for specified parameters, and tenfold cross-validation was used to evaluate the performance of the XGBoost. The grid search made XGBoost achieve a good generalizability but also consumed more calculation resources and storage space. In addition, XGBoost model fit the range of normal values more stably, but the ARIMA model was slightly better than the XGBoost model when fitting outliers (Fig. 8). This finding is mainly due to the following reasons: during ARIMA modeling, the best parameters were determined by the minimum CAIC and residual white noise of the training set, and the problem of overfitting was not considered. For the XGBoost model, to prevent overfitting, tenfold cross-validation and an early-stopping mechanism were used to select the best parameters. These factors increased the prediction performance of the XGBoost model but reduced the fitting effect of outliers. With these characteristics, our study applied it in prediction of the incidence of HFRS. We tried one-step forecasting and multistep XGBoost forecasting models to predict HFRS cases in mainland China. The results showed that the MAEs of the one-step and multistep XGBoost models were 132.055 and 173.403 respectively, which were 28.76 and 33.27 % lower than that of ARIMA model. The MAPE values were 12.353 and 15.615, which were 33.45 and 48.16 % lower than that of the ARIMA model. The RMSEs were 178.547 and 223.187, which were 28.37 and 26.29 % lower than that of ARIMA model.

As predicted, the one-step prediction accuracy of the two models was better than the multistep prediction accuracy. From the perspective of predicting infectious diseases, each predicted value of one-step prediction is obtained from the actual value, and it is unrealistic to predict diseases that have not occurred. Multistep prediction uses the previous prediction value as input to predict the next value, which will produce cumulative errors, but it has practical significance for predicting infectious diseases that have not occurred. The results indicated that the proposed one-step and multistep XGBoost model can significantly improve the accuracy of the overall prediction. The value of Theil’s U also proved this finding. From the perspective of the prediction accuracy and prediction stability, the XGBoost model is suitable for HFRS prediction tasks. In other words, by integrating the prediction results of multiple regression trees, the XGBoost model can achieve better prediction results than the ARIMA model in the one-step forecasting and multistep forecasting.

## Conclusions

In this paper, we built a seasonal ARIMA model and XGBoost model to conduct one-step and multistep prediction of the number of HFRS cases in mainland China for 2004 to 2018. The multistep XGBoost prediction model showed a much better prediction accuracy and model stability than the multistep ARIMA prediction model. The XGBoost model performed better in predicting complicated and nonlinear HFRS data. Additionally, a multistep prediction model has more practical significance than one-step prediction for forecasting infectious diseases.

## Supplementary Information

**Additional file 1.** Monthly cases of hemorrhagic fever with renal syndrome in Mainland China from 2004 to 2018.

## Data Availability

The datasets generated and/or analysed during the current study are available in Additional file 1 (http://www.nhc.gov.cn). The datasets used and/or analysed during the current study are also available from the corresponding author on reasonable request.

## Abbreviations

HFRS: Hemorrhagic fever with renal syndrome
CDC: Center for Disease Control and Prevention
ARIMA: Autoregressive integrated moving average
XGBoost: Extreme gradient boosting
CART: Classification and regression tree
ADF: Augmented Dickey-Fuller
ACF: Autocorrelation function
PACF: Partial autocorrelation function
CSS: Conditional sum of squares
CAIC: Corrected Akaike Information Criteria
ME: Mean error
RMSE: Root mean squared error
MAE: Mean absolute error
MPE: Mean percentage error
MAPE: Mean absolute percentage error
MASE: Mean absolute scaled error
ACF1: Autocorrelation of errors at lag 1
